# Emotional reactions to deviance in groups: the relation between number of angry reactions, felt rejection, and conformity

**DOI:** 10.3389/fpsyg.2015.00830

**Published:** 2015-06-15

**Authors:** Marc W. Heerdink, Gerben A. van Kleef, Astrid C. Homan, Agneta H. Fischer

**Affiliations:** ^1^Department of Social Psychology, University of Amsterdam, AmsterdamNetherlands; ^2^Department of Work and Organizational Psychology, University of Amsterdam, AmsterdamNetherlands

**Keywords:** emotion, social influence, conformity, social exclusion, group processes, deviance

## Abstract

How many members of a group need to express their anger in order to influence a deviant group member’s behavior? In two studies, we examine whether an increase in number of angry group members affects the extent to which a deviant individual feels rejected, and we investigate downstream effects on conformity. We show that each additional angry reaction linearly increases the extent to which a deviant individual feels rejected, and that this relation is independent of the total number of majority members (Study 1). This felt rejection is then shown to lead to anti-conformity unless two conditions are met: (1) the deviant is motivated to seek reacceptance in the group, and (2) conformity is instrumental in gaining reacceptance because it is observable by the majority (Study 2). These findings show that angry reactions are likely to trigger anti-conformity in a deviant, but they are also consistent with a motivational account of conformity, in which conformity is strategic behavior aimed at gaining reacceptance from the group.

## Introduction

Accumulating research illustrates that people are greatly influenced by other people’s emotional expressions (Van Kleef et al., 2011). Most of this work has examined how a single person’s emotional displays affect the perceptions, feelings, and behaviors of another person in dyadic interactions (e.g., Hatfield et al., 1994; Clark et al., 1996; Knutson, 1996; Hess et al., 2000; Van Kleef et al., 2004). However, people spend much of their social life in groups, for instance in work teams, in groups of friends, in school classes, and in sports teams. Compared to dyadic interactions, the potential number of emotional expressions is greater in groups, and such expressions might jointly influence individual group member’s cognitions, emotions, and behavior (Heerdink et al., 2013).

Groups are seldom unanimous, however, which implies that an increased number of emoters allows for greater variability of displayed emotions. In the present paper, we examine how multiple emotional expressions jointly influence a focal group member’s behavior. More specifically, we focus on the number of individuals that express anger. Is a single angry group member sufficient to influence a focal individual or should more group members express anger? To answer this question, we build on work on majority size and social influence in groups (e.g., Latané, 1981; Bond, 2005). We predict that the more group members react with anger to a focal individual, (1) the more this individual will feel rejected and (2) the greater the social influence, as reflected in conformity to the majority’s position. We tested these predictions in two experiments in which we employed a simulated majority influence paradigm.

## Expressions of Anger as Tools of Social Influence

People feel angry when they blame another person for an event that is incongruent with their goals (Lazarus, 1991). Anger is typically expressed when people intend to change the other person’s behavior to resolve this incongruence (Averill, 1982; Fridlund, 1994). Thus, expressing anger is functional (at least, from the expresser’s point of view) to the extent that it leads to behavioral change in the observer (Fischer and Roseman, 2007; Van Kleef, 2009). For instance, it has been shown that expressions of anger can help to extract concessions from negotiation partners (Van Kleef et al., 2004), that a teacher’s angry expressions can increase a student’s learning performance (Van Doorn et al., 2014), and that leaders’ displays of anger can enhance follower motivation and performance (Damen et al., 2008; Van Kleef et al., 2010).

Within a group context, angry expressions can be seen as cues of imminent exclusion, because the expression of anger and other types of hostility typically precedes the exclusion of deviants (Schachter, 1951). Anger may further signal rejection because it draws attention to the unacceptability of an individual’s deviant behavior, and by extension, of the individual him- or herself (Heerdink et al., 2013, 2015). Supporting this reasoning, Heerdink et al. (2013) demonstrated that when multiple group members unanimously expressed anger about a deviant person’s behavior, the deviant individual felt rejected by the group.

Maintaining a sense of belonging is a fundamental human need (Baumeister and Leary, 1995). Feeling rejected therefore potentially triggers behavior aimed at restoring the connection with other people (e.g., Williams et al., 2000; Romero-Canyas et al., 2010; DeWall and Richman, 2011). Because conformity signals good group membership (Hollander, 1960) and facilitates collective goal pursuit by restoring group cohesion (Festinger, 1950; Jetten and Hornsey, 2014), conformity is an effective way for deviants to seek reacceptance when they feel rejected as a consequence of other group members’ angry reactions. Congruent with this idea, Heerdink et al. (2013) found that participants who felt rejected by their (unanimously) angry fellow group members were likely to conform to the group norm, provided that their conformity could facilitate their reconnection with the group. What is unclear, however, is how many angry group members it takes to enforce such social influence.

## Number of Angry Expressions, Feelings of Rejection, and Conformity

Insights about the relation between the number of angry reactions and the degree to which the deviant will conform can be gleaned from more general theories about the cumulative influence of multiple influence sources. For instance, Social Impact Theory (SIT; Latané, 1981) describes the mathematical relation between the number of influencer sources and their influence on an individual person. The theory predicts that social influence increases as the number of influencers increases. Additionally, SIT proposes that the relation between the number of influencers and their social impact (everything else being equal) follows a power law, which implies that each additional influence source is expected to add to the total social influence, but the increase is smaller than for the previous influence source.

The consequences of varying numbers of social influence sources have primarily been investigated in the context of majority and minority influence (e.g., Latané and Wolf, 1981; Bond, 2005). For instance, a meta-analysis of 115 conformity studies shows that the number of influencers is indeed positively (albeit not very strongly) associated with the degree of social influence that is engendered (Bond, 2005). Furthermore, Bond (2005) found that, despite showing a slightly better fit to the data, SIT’s power function did not yield a significantly improved prediction over a linear model when majority sizes of 1 were excluded, indicating that the relationship between number of influencers and social impact is most parsimoniously represented as a linear function.

Some research has found that social exclusion is similarly dependent on the number of excluders, but the evidence shows that the direction of this effect may additionally depend on the type of exclusion (active versus passive). With regard to passive exclusion (e.g., ignoring), a recent meta-analysis of 98 Cyberball studies (Hartgerink et al., 2015) found that the ostracism effect in Cyberball is slightly smaller with three other players than with two other players, although the authors note that no studies directly comparing these two settings have been conducted, and the evidence for this difference was generally quite weak. Focusing on more active exclusion, DeWall et al. (2010) tested the relation between the number of group members who did not join in the social exclusion of a participant (e.g., by indicating their willingness to work with the participant) and the extent to which participants felt rejected. They found that felt rejection decreased as the number of accepting group members increased. Thus, social exclusion may decrease with the number of passive excluders, and increase with the number of active excluders.

Because angry reactions constitute an active type of rejection, we hypothesize that deviant group members feel more rejected the more fellow group members express anger about their deviance (H1). Given that feeling rejected motivates a desire to seek reacceptance, we predict that deviants conform more to the extent that they receive more angry reactions (H2), and that this relationship is mediated by felt rejection (H3). We conducted two experiments to test these hypotheses. In both studies, a simulated group interaction was set up in which the participants’ opinion was opposite to their fellow group members’ opinions. Thus, the situation represented a majority influence situation, in which the participant had a deviant position. The majority then responded emotionally to the participants’ deviance. We used neutral to mildly happy reactions as the non-angry reactions in our studies. Previous research suggests that expressing some happiness is the ‘default’ in positive social interaction (e.g., Fridlund, 1991; Hinsz and Tomhave, 1991; Jakobs et al., 2001), and we reasoned that it would therefore the most ‘normal’ reaction in such a group setting.

## Study 1

In Study 1, we systematically varied two factors: the size of the majority (i.e., the total group size minus the deviating participant) and the number of angry reactions from majority members to test whether the number of angry reactions uniquely affects felt rejection, or whether this depends on the total number of majority members. Varying the number of angry reactions to deviance within a group means that the number of non-angry reactions also varies. If, as we hypothesized, felt rejection and subsequent conformity increase with the number of angry reactions, this relation should be found independently of the number of non-angry reactions. Thus, majority size should not moderate the effect of angry reactions on felt rejection or conformity. To separate the effects of majority size from those of the emotional reactions, the experiment was set up in such a way that, independent of their emotional reactions, all majority members disagreed with the participant and agreed with each other with regard to their position in the debate.

For the sake of brevity, we use the notation M|A to refer to experimental conditions. M denotes majority size, and A refers to the number of angry reactions. The number of non-angry reactions may be calculated as M – A. Thus, a participant in condition 4|1 was confronted with a majority of four, received one angry reaction, and three (i.e., 4 – 1) mildly happy reactions. Finally, the letters M or A are used when referring to all levels of a manipulation: 3|A refers to all Majority size 3 conditions (3|0, 3|1, 3|2, and 3|3), and M|1 to all conditions with one angry reaction (2|1, 3|1, and 4|1).

### Method

#### Participants and Design

Three-hundred and seventy first-year Psychology students participated in the study as part of an obligatory, 2-h mass testing session that took place at the beginning of the academic year. Participants in the current study were part of two groups of around 225 students each, who were simultaneously seated in front of a computer (separated with dividers) in a large room. Thus, the setting rendered it plausible that the participant would interact with other participants during the study. The majority of tasks preceding the current study were personality questionnaires (and all unrelated to the current study), but there were slight differences in the number (six and eight) and content of the tasks between the two groups. Details may be obtained from the first author.

Of the 370 participants, 56 participants were excluded because their open-ended responses indicated that they doubted the veracity of the simulated interaction^1^. Expression of doubts was not predicted by the manipulations. An additional 34 participants were excluded because they misremembered the number of group members they interacted with, suggesting that they had not paid sufficient attention to the instructions. Misremembering the number of fellow group members was more likely as the number of fellow group members increased (*OR* = 3.53, Wald’s *z* = 3.85, *p* < 0.001)^2^. The final sample thus consisted of 280 participants (75 male, *M*_age_ = 19.70, range 18–28). Participants were randomly assigned to a condition of the Majority Size (2, 3, or 4) × Angry Reactions (0, 1, 2, 3, or 4) design (logically impossible conditions in which the number of angry reactions exceeded the majority size were, of course, omitted); the distribution over conditions is displayed in **Table 1**. The study was carried out in accordance with APA regulations and approved by the IRB at the University of Amsterdam.

**Table 1:** Number of participants conforming and total number of participants in each condition (Study 1).

		Number of angry reactions				
		0	1	2	3	4
Number of majority members	2	5/27 (18.5%)	9/23 (39.1%)	14/26 (53.8%)		
	3	8/24 (33.3%)	14/29 (48.3%)	7/21 (33.3%)	13/27 (48.1%)	
	4	5/21 (23.8%)	11/23 (47.8%)	10/19 (52.6%)	10/23 (43.5%)	8/17 (47.1%)

*Conditions are based on majority size and the number of angry reactions received by the participant. Cell sizes vary due to random assignment to conditions. Three cells are empty because there cannot be more angry reactions than there are members of the majority.*

#### Materials and Procedure

The experiment was introduced as having two goals: to investigate the opinions of students on a number of study-related issues, and to determine the efficiency of a newly developed discussion technique called the ‘one-shot discussion,’ which was defined as a discussion in which every participant gets one chance to make a statement.

##### Initial opinion

Participants first provided their opinion about nine student-related issues. Among these was the issue that would be used later in the group discussion (the focal issue): the percentage of the study materials in the first and second years of the Bachelor’s program that should consist of journal articles relative to books. Responses could be made using a slider that ranged from 30 to 70% so as to anchor responses around 50%, which was used as a reference point to determine the group norm (see ‘Deviance Manipulation’ below). Alternatively, participants could enter a whole number between 0 and 100 in a separate box.

##### Majority size manipulation

Participants then learned that they would be participating in a one-shot discussion on one of the student-related issues. The program simulated connecting to a number of fellow participants in the mass testing session. Depending on the majority size condition, the connection routine ‘discovered’ two to four other participants before proceeding to the next stage. Thus, total group sizes for the group discussion (including the participant) varied from three (in the 2|A conditions) to five (in the 4|A conditions).

##### Deviance manipulation

The next screen indicated that the ‘articles vs. books’ issue had been selected, and participants were presented with information that indicated that their opinion deviated from the group norm. The group norm was manipulated by showing the answers that the fellow group members had supposedly given, and were drawn from one of two sequences. For the 206 participants (73.6%) whose initial answer fell below 50%, the majority’s answers were shown to have been 68, 90, 75, and 85 (‘many articles’ group norm); for the remaining participants, who had originally answered more than 50%, the corresponding majority answers were 32, 10, 25, and 15 (‘few articles’ group norm). The number of answers shown corresponded with the Majority size condition. For instance, participants in the 2|A conditions who preferred less than 50% of the study materials to consist of journal articles learned that their first fellow group member had answered 68%, and the second 90%. (No more answers were shown, because there were no more group members in this condition).

##### Angry reactions manipulation

The next phase was the group discussion, which contained the manipulation of the group’s angry reactions. In the group discussion, the group members would each send a successive statement about their opinion to the others. The participant learned that s/he would be the last to state their opinion to the others.

The statements contained arguments and were framed in either a mildly happy or angry way. Four arguments were used for each of the two possible group norms (more articles or more books). The emotional tone of the statements was manipulated by means of emotional words such as ‘annoys me’/‘makes me angry’; words with strong emotional overtones such as ‘ridiculous’; and happy versus angry emoticons, that is, :) or >:(. To avoid a confound between majority size and the number of presented arguments, the statements were written in such a way that all participants read all four arguments. Thus, one of the majority members in the 3|A conditions, and both majority members in the 2|A conditions used two arguments in their statements. Example statements can be found in **Table 2**.

**Table 2:** Example statements sent by the simulated group members during the simulated group interaction (Study 1).

Norm: many articles		Group norm: few articles	
Mild happy	Angry	Mild happy	Angry
Later in our study, we’ll have to read those articles anyway, so I think it’s better to get used to that style as soon as possible..	It’s ridiculous that we have books for absolutely everything! We’ll be reading those articles later in our study anyway, so doesn’t it make sense to get used to that style as soon as possible?	I often don’t see the connections between articles and other research, so I prefer a book.. :)	In articles it’s often totally unclear how it connects to other research, so having so many articles won’t help us in any way!
For my part, we’ll just do almost everything using journal articles, it’s much cheaper!:)	For my part, we’ll just do almost everything using journal articles, it’s much cheaper! Not everyone can afford those books so easily!!! >:(	For my part, we’ll just do almost everything using books, I find it handy to have a good reference on the bookshelf!	For my part, we’ll just do almost everything using books, it really annoys me that some people think it’s a good idea to first print everything and then throw it away, rather than investing in something durable >:(
Journals are much more up-to-date than books, right? Seems better to me to get an idea of what’s happening in psychology directly from the start!	Journals are much more up-to-date than books, right? I find it really stupid to waste our time by learning about obsolete theories..	I’d rather have one book that just contains everything instead of having to look for an article again and again..	Ridiculous idea, it’s often impossible to even find an article.. please give me a book that just contains everything!
Everything has already been said really, but isn’t it just better to read the original instead of what someone else thinks about that?	Indeed, don’t you just want to read the original instead of how some book writer interprets that??	I’m also against articles, they’ve been written only so that it suits the author, I think a book is much more objective!	I’m also against articles, theres no point in reading only that which happens to suit the author?! A book is much more objective..

*These statements were used in the conditions with four majority members. Depending on the group norm (which was manipulated to be opposite to the participant’s initial opinion) and the assigned number of angry reactions, one statement from each row was sent to the participant.*

After having received all the simulated group members’ statements, participants were asked to write a statement themselves. These statements were not analyzed; rather, we used them to estimate whether participants doubted the reality of the situation (see ‘Participants and Design’ above). After writing and sending their statement, participants were given 30 s to read and study all the statements that had been made in the discussion.

##### Conformity measure

Next, the participants read that a student body had developed a proposal related to the focal issue. This proposal was manipulated to be consistent with the group norm (and therefore opposed to the participant’s position): The student body proposed to increase the percentage of journal articles to a minimum of 75% when the group norm was ‘many articles,’ or to reduce the percentage to a maximum 25% when the group norm was ‘few articles.’ Then, participants were asked to vote. Because the framing of the proposal was consistent with the group norm, a higher proportion of votes for the proposal reflected more conformity.

##### Acceptance/rejection scale

Following four filler items that asked about the extent to which the discussion had been satisfactory, felt acceptance/rejection was measured using the four-item 7-point bipolar scale used by Heerdink et al. (2013), e.g., “I feel rejected by the group” (1 = *not at all*, 7 = *very much*; α = 0.64).

##### Manipulation checks

Two items checked whether participants perceived the group norm accurately (e.g., “My fellow group members prefer books rather than journal articles,” *r* = -0.82, *p* < 0.001). These items were embedded in a questionnaire that checked participants’ impressions of the discussion (e.g., the extent to which they thought the others agreed with each other).

To check the manipulation of majority size, participants were then asked to indicate with how many people they had interacted (0–4). Thirty-four participants misremembered majority size, and they were excluded from the analyses.

Three questions were used to check the manipulation of angry reactions. A first question asked whether or not the other group members had expressed anger during the interaction (yes or no). A second question asked how many of their fellow group members had expressed anger (0–5). A third question asked how much anger their fellow group members had expressed (1 = *not at all*, 7 = *very much*).

Finally, participants were asked the open-ended question, “Did you notice anything abnormal, strange, or that the experimenters should know about (e.g., apparatus failure)?”

##### Debriefing

At the end of the computerized mass-testing session, participants received a booklet that contained the debriefing for all experiments included in the session. The debriefing contained a description of the purpose of the study, explained the aspects of the experiment that had been simulated, and provided an e-mail address where more information could be obtained.

### Results

#### Analytic Strategy

Unless otherwise stated, analysis of each dependent variable began by fitting a full (linear regression) model with the Majority Size × Angry Reactions interaction and main effects as linear predictors^3^. Because less immediate influence sources are less able to engender social impact (Latané, 1981), we controlled for the immediacy of the other group members as a source of social influence by including a measure of social distance as a covariate. It was calculated as the numerical distance between a participant’s initial opinion and the group norm (the average of the fellow group members’ answers), and reflects the extent to which the participant occupied a deviant position in the group. We refer to this variable as *level of deviance*.

After fitting the full model, this model was simplified using standard model simplification procedures: Non-significant predictors were eliminated step-by-step, starting with the more complex terms (i.e., interactions before main effects). The predictive power of the simplified model was re-assessed after each elimination. The reported, final models are the simplest models (i.e., fewest predictors) that do not sacrifice predictive power relative to the full model. That is, a model comparison yields a non-significant (*p* >= 0.050) difference between the full and the final model.

#### Manipulation Checks

Analysis of the group norm manipulation check indicated that participants accurately remembered the group norm in their group. Participants perceived their fellow group members to be more in favor of articles when the group norm had been ‘many articles’ compared to ‘few articles,’ β = 2.03, *t* = 33.78, *p* < 0.001. No other effects were retained in the final model, *R*^2^ = 80.4%, *F*(1,278) = 1141.22, *p* < 0.001. The group norms were also perceived as close to the relevant extremes of the 7-point scale (1 = *more books*, 7 = *more articles*) in both the ‘many articles’ groups (*M* = 6.07, SD = 0.89) and the ‘few articles’ groups (*M* = 2.05, SD = 0.85). Thus, the group norms were clear to the participants.

The three angry reactions manipulation checks converged in showing that the angry reactions manipulation had been successful. First, a logistic regression indicated that the likelihood of reporting that fellow group members had expressed anger increased as the number of angry reactions increased, *OR* = 2.77, Wald’s *z* = 7.61, *p* < 0.001. Second, the reported number of angry reactions increased linearly as the manipulated number of angry reactions increased, β = 0.47, *t* = 11.77, *p* < 0.001 [*R*^2^ = 33.3%, *F*(1,278) = 138.64, *p* < 0.001]. Third, we found that with every extra angry reaction, participants reported that their fellow group members had expressed more anger, β = 0.47, *t* = 11.59, *p* < 0.001 [*R*^2^ = 32.6%, *F*(1,278) = 134.37, *p* < 0.001]. No other effects were retained in any of the three final models. Together, these strong and positive effects indicate that the angry reactions manipulation was successful.

#### Acceptance/Rejection

We found a small but reliable effect of angry reactions on felt rejection, indicating that participants felt more rejected as the number of angry reactions increased, β = 0.15, *t* = 3.22, *p* = 0.001. Moreover, the covariate was significantly related to felt rejection: participants felt more rejected as they were more deviant, β = 0.14, *t* = 2.33, *p* = 0.020. No other predictors were retained in the final model [*R*^2^ = 5.4%, *F*(2,277) = 7.91, *p* < 0.001]. The results thus support H1: Felt rejection increased as the number of angry reactions increased, independent of the size of the majority.

#### Conformity

Logistic regression on participants’ votes (coded so that positive regression coefficients indicate an increase in the likelihood of conformity; see **Table 1** for the exact number of participants conforming in each condition) found a small effect of the number of angry reactions, indicating that conformity increased with the number of angry reactions, *OR* = 1.32, Wald’s *z* = 2.55, *p* = 0.011. The covariate was also significant, indicating that conformity was less likely to the extent that the participant initially disagreed more with the group, *OR* = 0.41, Wald’s *z* = -5.58, *p* < 0.001. Thus, the data support H2 that deviant individuals are more likely to conform when more of their fellow group members respond with anger to their deviance.

#### Mediation Analysis

To test whether the effect of angry reactions on conformity could be explained by participants’ feelings of rejection, we conducted a mediation analysis. Using logistic regression, the participants’ decision was regressed on level of deviance (covariate), angry reactions, and the interaction between majority size and felt rejection. Model simplification dropped the majority size manipulation from the model. As before, we found that conformity was less likely to the extent that participants were more deviant, *OR* = 0.41, Wald’s *z* = -5.41, *p* < 0.001. Unexpectedly, and contrary to H3 that feeling rejected would explain the positive effect of angry reactions on conformity, we found marginally significant evidence that the likelihood of conformity was *reduced* to the degree that participants had felt rejected, *OR* = 0.78, Wald’s *z* = -1.77, *p* = 0.077. Additionally, the number of angry reactions remained a significant and positive predictor of conformity, *OR* = 1.37, Wald’s *z* = 2.81, *p* = 0.005.

When the coefficients obtained from the mediation analysis are compared to those from the analysis of conformity above, a small increase in the regression coefficient for the number of angry reactions may be observed (from *OR* = 1.32 to *OR* = 1.37). This indicates a potential suppressor effect (MacKinnon et al., 2000), which means that angry reactions may have had two simultaneous effects: a direct effect of angry reactions that increased conformity; and an indirect effect of angry reactions, through felt rejection, which reduced conformity (cf. Hayes, 2009). To test this possibility, the strength of the indirect effect of angry reactions on conformity through felt rejection was estimated using bootstrapping (*R* = 50,000 resamples). There was indeed some evidence for an indirect, conformity-reducing path, *OR* = 0.963, 95% bias-corrected and accelerated confidence interval (95% BC_a_ CI): [0.904, 0.999], uncorrected two-tailed *p* = 0.069. Although this indirect effect was quite small, it suggests that the likelihood of conformity was simultaneously increased by more angry reactions, and decreased by the felt rejection that was caused by these angry reactions.

### Discussion

Study 1 showed that deviant individuals felt more rejected, and conformed more, the more their fellow group members responded with anger to their deviant position, supporting H1 and H2, respectively. Moreover, as expected, these relations were not moderated by the size of the majority. However, the effect of angry reactions on conformity was not mediated by felt rejection. In fact, contrary to H3, the indirect effect of angry reactions on conformity was negative, suggesting that angry reactions reduced conformity through felt rejection. This unexpected result led us to consider more closely what might be driving the relationship between felt rejection and conformity. Previous work suggests that responses to exclusion depend on the prospect of being reaccepted (DeWall and Richman, 2011). Thus, whether people conform after feeling rejected by others may depend on two conditions (see also Matschke and Sassenberg, 2010; Romero-Canyas et al., 2010; Heerdink et al., 2013). First, the rejectee should be motivated to be reaccepted. Second, there should be an actual possibility of reacceptance by the group through conformity (Heerdink et al., 2013). That is, deviants should be more likely to conform when changing their position toward the group norm is instrumental in eliciting (re-)acceptance.

With regard to the first condition, the data of Study 1 showed that conformity was less likely to the extent that participants disagreed more with the majority of their group. This is consistent with classic work showing that people are more influenced by similar others (Festinger, 1950; Latané, 1981). Because similarity increases interpersonal attraction (Montoya et al., 2008), less deviant participants may have felt more attracted to their groups than more deviant individuals. As a result, they may have been more motivated to seek reacceptance, which helps explain why conformity was higher among less deviant participants.

The finding that feeling rejected was associated with decreased conformity may indicate that conformity was not perceived as instrumental to gaining reacceptance in Study 1. Indeed, it has been argued that social exclusion is likely to trigger anti-social behavior if there is no real prospect of reacceptance (DeWall and Richman, 2011). It is possible that the operationalization of conformity in terms of voting behavior may have inspired a sense of anonymity among participants, because votes are often anonymous. Thus, participants may have inferred that the majority would not observe their conformity and therefore would not reaccept them, even if they conformed. This implies that we may find a different effect if the majority can observe the deviant’s conformity. We examined this possibility in Study 2.

## Study 2

In Study 2, we investigated whether the effect of majority anger on a deviant individual’s conformity depends on the deviant’s sense of anonymity. For this purpose, we included a manipulation of whether the participants’ final decisions were private (as in Study 1) or public. We hypothesized that there would be a more positive association between felt rejection and conformity if the decision was public rather than private (H4).

We further explored whether the initial level of deviance of the participant served as an additional moderator, such that the anonymous or public nature of the final decision would only have an effect on those participants who are not too far removed from the group norm (i.e., those who are relatively less deviant). Participants who are very deviant from the group should be less attracted to their groups (Montoya et al., 2008), which may lower the motivation to seek reacceptance. Thus, we explored whether our data fit the idea that feeling rejected increases conformity only when two criteria are met: (1) the level of deviance is relatively small, and (2) conformity is visible to the group (i.e., under public, but not under private voting).

### Method

#### Participants and Design

Two-hundred and forty-seven first-year Psychology students participated in the study, which was part of a similar mass testing session as Study 1. Again, participants came from two different groups that differed in the number (nine and eight) and content of the preceding tasks (which were, again, primarily personality questionnaires and unrelated to the current study). Details about these tasks may be obtained from the first author. Participants whose responses to the open questions suggested doubt about the reality of the simulated interaction or computer problems (*n* = 11)^4^, and participants who misremembered the number of group members they had interacted with (*n* = 19) were excluded, resulting in a sample of 217 participants (64 male, *M*_age_ = 19.43, range 18–27). Failing these checks was not predicted by the manipulations. All participants interacted with a majority of three^5^, and they were randomly assigned to one of the conditions of the Angry Reactions (0, 1, 2, or 3) × Decision Context (public or private) design. The distribution over conditions is displayed in **Table 3**. The study was carried out in accordance with APA regulations and approved by the IRB at the University of Amsterdam.

**Table 3:** Number of participants conforming and total number of participants in each condition (Study 2).

		Number of angry reactions			
		0	1	2	3
Reponse context	Public	18/27 (66.7%)	16/25 (64.0%)	15/28 (53.6%)	12/24 (50.0%)
	Private	13/27 (48.1%)	14/30 (46.7%)	17/30 (56.7%)	11/26 (42.3%)

*Conditions are based on response context and the number of angry reactions received by the participant. Cell sizes vary due to random assignment to conditions.*

#### Materials and Procedure

Study 2 was similar to Study 1, and revolved around the same issue (the percentage of journal articles versus books). In addition to the procedural changes described below, we made two minor changes. First, the statements sent by the simulated participants were slightly edited to be more consistent in terms of wording and length (**Table 4**). Second, one of the angry reactions manipulation checks (the question “How many of your fellow group members had expressed anger?”) was dropped for reasons of economy.

**Table 4:** Statements sent by the simulated group members during the simulated group interaction (Study 2).

Norm: many articles		Group norm: few articles	
Mild happy	Angry	Mild happy	Angry
Later in our study, we’ll have to read those articles anyway, so I think it’s convenient to get used to that style as soon as possible..	We’ll be reading those articles later in our study anyway, so we should get used to that style as soon as possible, right? It’s ridiculous that we have to use books first!	In articles, the connections to other research are not as clear as in books so I’d prefer books..	In articles it’s often totally unclear how it even connects to other research, so it’s ridiculous to do away with books for that
For my part, we’ll just do almost everything using journal articles, it’s much cheaper!:)	For my part, we’ll just do almost everything using journal articles, it’s much cheaper! Not everyone can afford those books so easily!!! >:(	Printing articles costs a lot of paper and ink, and you throw them away anyway, so books are much better for the environment. Much more sustainable:)	Using articles instead of books is nothing but pollution!! Do you know how much ink and paper that takes? And we throw them away anyway, so they’re just worthless >:(
Journals are much more up-to-date than books, right? If we use journal articles we get an idea of what’s happening in psychology directly from the start!	Journals are much more up-to-date than books, right? it really irritates me to have to learn about all kinds of obsolete theories first	I’d rather have one book that just contains everything instead of having to look for individual articles on the internet..	It’s often completely impossible to find an article with these half-broken search engines, so I would find it really super irritating to have to read so many articles..

##### Deviance manipulation

The initial opinion measure was modified so that the slider ranged from 10 to 70%, and the group norm was now determined using the critical value of 40%. Participants whose initial opinion was less than 40% interacted with a group that endorsed the ‘many articles’ group norm, and the remaining participants with a group in which ‘few articles’ was the group norm. The fellow group members’ opinions (which constituted the deviance manipulation) were also adjusted so that both group norms were equally far away from the critical value of 40%. The sequences were 52–69–60 for the ‘many articles’ group norm, and 28–11–20 for the ‘few articles’ group norms. The percentage of participants interacting with a group with the ‘many articles’ group norm (73.3%) was comparable to that in Study 1 (73.6%).

##### Decision context manipulation

For participants in the private decision condition, the procedure was identical to that in Study 1. For participants in the public decision condition, the procedure differed in several ways. First, participants learned that they would have to explain their final decision to their fellow group members^6^. Second, after completing the discussion, a new instruction screen alerted participants that their decision would be visible to their fellow group members, and that they would need to write an explanation for their decision that would be sent to their fellow group members. The decisions would again be taken one-by-one, in the reverse order in which the statements had been written. Because the participants had always written the last statement, they would always be the first to take and explain their decision. This ensured that the participant would not be influenced by anything but the statements they had read during the discussion.

After participants had made their decisions, the program simulated a connection failure, and subsequently the connection timed out. The purpose of this procedure was to avoid having to present any simulated decisions/explanations to the participant, which could potentially alter the participants’ responses in the questionnaire.

##### Decision context manipulation check

To check whether the decision context manipulation (public vs. private) was had been successful, participants were asked to indicate their agreement with the statement “I could take my decision anonymously” on a 7-point scale (1 = *not at all*, 7 = *very much*). This item was added to the questionnaire that also contained the items that checked the perception of the group norm.

### Results

#### Analytic Strategy

We analyzed the data using the same general strategy as in Study 1. In this case, the full model contained the Angry Reactions × Decision Context interaction and main effects as linear predictors, and level of deviance was again included as a covariate. Once again, the reported final model is the simplest model that does not sacrifice predictive power compared to the full model.

#### Manipulation Checks

Analysis of the group norm manipulation check indicated that participants had perceived the group norm correctly. Participants perceived the norm to be much closer to the ‘journals’ end of the scale (from 1 = *more books* to 7 = *more journals*) when the group norm was ‘many articles’ (*M* = 6.10, SD = 0.80) rather than ‘few articles’ (*M* = 1.86, SD = 0.86), β = 2.07, *t* = 33.90, *p* < 0.001. No other predictors were retained in the final model, *R*^2^ = 84.2%, *F*(1,215) = 1149.13, *p* < 0.001. This strong effect shows that the group norms were clear.

The manipulation check for decision context was influenced by whether the decision was private or public. The decision context effect was small and showed that, as intended, participants in the private decision condition (*M* = 5.89, SD = 1.25) reported that they could take their decision more anonymously than participants in the public decision condition (*M* = 5.39, SD = 1.52), β = 0.36, *t* = 2.65, *p* = 0.009. The final model contained no other predictors, *R*^2^ = 3.2%, *F*(1,215) = 7.03, *p* = 0.009.

Analysis of the manipulation checks for angry reactions showed that this manipulation also worked as intended. First, a logistic regression analysis on the question of whether the other group members had expressed anger indicated that more angry reactions increased the likelihood of answering this question affirmatively, *OR* = 2.64, Wald’s *z* = 6.18, *p* < 0.001. Second, the other group members were perceived to be more angry as the number of angry reactions increased, β = 0.44, *t* = 8.06, *p* < 0.001 [*R*^2^ = 23.2%, *F*(1,215) = 64.89, *p* < 0.001]. No other effects were retained in the final models. These strong effects show that the angry reactions manipulation was successful.

#### Acceptance/Rejection

We predicted that participants would feel more rejected as they received more angry reactions. The final model supported this prediction, *R*^2^ = 6.4%, *F*(2,214) = 7.35, *p* = 0.001. The effect of the number of angry reactions was small and shows that as the number of angry reactions increased, participants felt more rejected, β = 0.15, *t* = 2.52, *p* = 0.012. In addition, as in Study 1, participants felt more rejected when they were more deviant, β = 0.18, *t* = 2.65, *p* = 0.009. No other effects were retained in the final model.

#### Conformity

Logistic regression on the decisions made by participants (see **Table 3** for the exact number of participants conforming in each condition) revealed that the predicted interaction between angry reactions and decision context could be dropped from the model without losing predictive power [Δχ^2^(1) = 0.17, *p* = 0.681]. Thus, H4 that felt rejection would increase conformity in a public decision context was not supported. Further simplification of the model showed that the manipulations were all dropped from the model. However, replicating Study 1, the results did show that the participant’s level of deviance moderately predicted conformity: being more deviant decreased the likelihood of conformity, *OR* = 0.45, Wald’s *z* = −4.99, *p* < 0.001.

**FIGURE 1:**
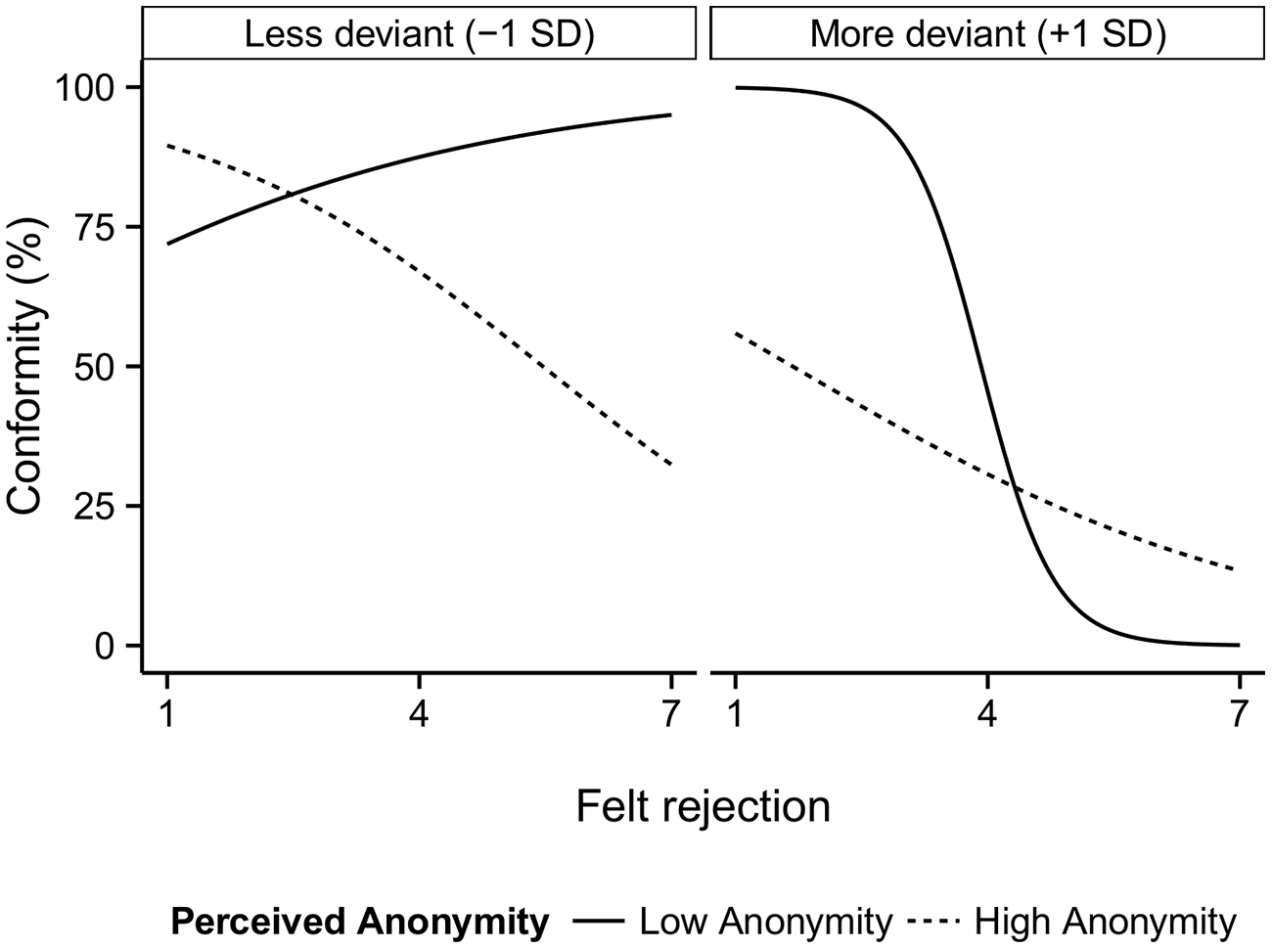
**Plot of the predicted values from the three-way Felt Rejection × Anonymity × Level of Deviance interaction on conformity.** The panels show the differences between relatively less deviant and relatively more deviant group members. The line types are based on the anonymity manipulation check, and show the different relation between felt rejection and conformity depending on the subjective anonymity of the decision (low and high anonymity, or 2 and 6 on the 7-point scale, respectively).

Interestingly, subsequent exploratory analyses provided support for the idea that the relationship between felt rejection and conformity is contingent upon the decision context as well as the amount of initial deviance of the participant. In these analyses, we increased our statistical power by using the anonymity manipulation check as a predictor instead of the decision context manipulation. A model that included the three-way Felt Rejection × Anonymity × Level of Deviance interaction significantly improved the prediction of conformity, relative to the model that contained only felt rejection and level of deviance as predictors [Δχ^2^(5) = 12.17, *p* = 0.033]. A plot of this three-way interaction (*OR* = 1.63, Wald’s *z* = 2.43, *p* = 0.015; see **Figure 1**) indicates that the relation between felt rejection and conformity was generally negative. Only for relatively less deviant participants who did not feel anonymous, the relation between felt rejection and conformity was more positive.

#### Indirect Effect

Study 1 indicated that angry reactions produced two competing effects: one direct, that increased conformity; and one through felt rejection, that decreased conformity. Not finding a relation between angry reactions and conformity may thus simply indicate that the positive and negative effects of angry reactions were canceling each other out (cf. Hayes, 2009). Thus, even in the absence of a main (total) effect, it is recommended to test for an indirect effect (Hayes, 2009).

We tested this indirect effect as in Study 1. First, we tested the relation between felt rejection and conformity, and whether this relation depended on decision context. Consistent with the existence of an indirect path, conformity was less likely to the extent participants felt more rejected, *OR* = 0.68, Wald’s *z* = -2.52, *p* = 0.012. In addition, as before, conformity was less likely to the extent participants were more deviant, *OR* = 0.48, Wald’s *z* = −4.61, *p* < 0.001. No main effects or interactions involving decision type were retained in the final model. Using bootstrapping (*R* = 50,000 resamples), we then directly tested the indirect path from angry reactions, through felt rejection, to conformity. The analysis supported the existence of this indirect effect: *OR* = 0.943, 95% BC_a_ CI: [0.854, 0.992], uncorrected two-tailed *p* = 0.024. No direct, conformity-increasing effect of angry reactions was found. Thus, the small, indirect, conformity-reducing effect from Study 1 was indeed replicated.

### Discussion

Study 2 replicated the finding that the more their fellow group members respond with anger to their behavior, the more deviant individuals feel rejected, and that this increased felt rejection subsequently decreases conformity. We hypothesized that in a public decision context, this felt rejection would be associated with increased conformity. Our results, however, show that the relation between felt rejection and conformity not only depends on the decision context, but also on one’s level of deviance: for relatively less deviant individuals who felt their decision would be public, we found evidence that the negative relation between felt rejection and conformity can reverse. The findings of Study 2 thus replicate and extend those of Study 1, and are consistent with the idea that conforming to the group requires both visibility of conformity, as well as a relatively lower level of deviance.

## General Discussion

Starting from the perspective that emotions are functional in regulating intragroup processes (e.g., Keltner and Haidt, 1999), and the observation that anger is expressed in order to change other people’s behavior (e.g., Fischer and Roseman, 2007), we raised the question of whether the number of angry reactions to a deviant group member influences felt rejection and conformity. In two studies, we found evidence for our prediction that deviant group members would feel increasingly rejected as the number of angry reactions from the majority increases, and we found this relation to be independent of the total size of the group (Study 1). Furthermore, we found that the felt rejection caused by these angry reactions led to anti-conformity, unless two criteria were met: the initial extent of deviance was relatively small (Studies 1 and 2), and conformity could be instrumental in gaining reacceptance (Study 2).

These studies not only provide insight into the dynamics of emotional influence within groups where multiple and different emotional expressions may occur, but they also illustrate the usefulness of studying the role of discrete emotional episodes in shaping intragroup processes. Existing research that focused on how affective phenomena impacts group outcomes (e.g., Barsade, 2002; Van Kleef et al., 2010) has primarily invoked the notion of emotional contagion, where one group member’s affective experiences infuse, or trigger similar affective experiences in another group member (Barsade, 2002). We complement this perspective by offering insight into how discrete emotional expressions (or episodes) affect group dynamics. Studying affective processes in this more fine-grained manner helps us to understand the circumstances under which emotional reactions to deviance may ignite, sustain, or help resolve intragroup conflict (e.g., Jehn, 1997).

Although we set out to demonstrate that more angry reactions may increase conformity, our findings generally show the opposite. As such, they speak to the recent increase in attention to conditions under which people resist pressures to conform and choose dissent instead (e.g., Packer, 2007; Packer and Chasteen, 2009; Jetten and Hornsey, 2014). Dissent is considered as an important factor in stimulating group creativity and avoiding group think (e.g., De Dreu and West, 2001; Nemeth et al., 2001). Jetten and Hornsey (2014) describe a number of motivations that may underlie dissent, including a desire to express individual difference [e.g., a desire for personal freedom of choice (e.g., Miron and Brehm, 2006) or seeking uniqueness (Hornsey and Jetten, 2004)], pro-social motivations (e.g., concern for the group when norms are perceived as harmful; Packer, 2007), and anti-social or destructive motivations that aim to harm the group (Jetten and Hornsey, 2014). How should anti-conformity in our studies be understood?

Given that felt rejection mediated the effect of increasing numbers of angry reactions on increased anti-conformity, interpretations in terms of a pro-social motivation fit the data less well than interpretations in terms of seeking individual difference, or anti-social motivations. With regard to the former, it is difficult to see why more rejected participants would be more concerned about the group’s well-being given that they are also more likely to leave the group when given the opportunity (Heerdink et al., 2013). Hence, the anti-conformity triggered by angry reactions is more easily interpreted as either an attempt to restore the freedom of choice (i.e., reactance; e.g., Miron and Brehm, 2006), or a more anti-social motivation to harm the group. An interpretation in terms of anti-social motivation is especially likely because rejection experiences have often been associated with antisocial behavior more generally (for a review, see Leary et al., 2006). For instance, in the previously discussed study by DeWall et al. (2010), participants who had been socially excluded by their peers allocated more hot sauce and administered longer blasts of loud noise to their rejecters. Furthermore, there is evidence that people who feel rejected are less inclined to cooperate with their groups (Twenge et al., 2007). Given that anti-conformity breaks the group’s consensus, which hinders coordinated goal pursuit (Festinger, 1950), the anti-conformity triggered by angry reactions may reflect an attempt to retaliate against the rejecters. The finding that angry reactions decreased conformity may thus reflect the joint impact of a desire for freedom of choice and anti-social motivations following rejection. The most important observation following this analysis, however, is that neither a motivation to restore the freedom of choice, nor anti-social motivation may be expected to result in the authentic type of dissent that has been found to be conducive to group functioning (Nemeth et al., 2001).

In addition to demonstrating a link between angry reactions and anti-conformity, we have found some evidence that the tendency for anti-conformity may be reduced if contextual factors both promote the motivation to remain a member of the group (e.g., under relatively less deviance, because similarity increases attraction; Montoya et al., 2008) and allow conformity to be instrumental in gaining reacceptance (e.g., when decisions are public). Previous work has indeed shown that in similar situations, angry reactions may actually elicit conformity from a deviant (Heerdink et al., 2013). The fact that we primarily observed anti-conformity may therefore reflect that the contextual factors that would promote conformity were simply not clear or strong enough in the current studies. Because we conducted the experiments with first year students, it is not unlikely that our participants’ overall degree of identification with their peers was quite low. Thus, their motivation to remain a member of their groups may have been simply insufficient (even when they were relatively less deviant) to completely reverse the relation between rejection and conformity, and show that a majority can indeed pressure a deviant individual into conforming by reacting with anger.

An interesting inconsistency between our findings and those from earlier majority influence research is that the size of the majority played no role in determining conformity (Study 1), despite the fact that majority size is often considered a determinant of conformity in the majority influence literature (e.g., Asch, 1956; Latané and Wolf, 1981; Bond, 2005). This may point to a similarity between the emotional influence process studied here and processes implicated in normative influence (Deutsch and Gerard, 1955). Normative influence stems from the power of the group to include or exclude individuals, and occurs when people change their opinion for fear of losing group membership (Deutsch and Gerard, 1955). By affecting one’s sense of acceptance versus rejection, angry reactions are likely to invoke the same motivations as underlying normative influence. Our finding that majority size did not influence conformity may thus indicate that majority size only plays a role when it is ambiguous to what extent deviance will lead to rejection. In this case, people may infer that they may be rejected if they stay deviant, which leads them to conform. In the current set of studies, information about contingent rejection was provided in the form of angry reactions, which may have disambiguated the situation. This explanation remains to be tested, however.

The direct and positive effect of angry reactions on conformity in Study 1 suggests that angry reactions may enhance informational influence as well. Informational influence occurs because the majority, due to its greater size, has a greater claim to objective reality than a single individual (Deutsch and Gerard, 1955). Informational influence thus occurs when a majority persuades an individual that a certain opinion or behavior is objectively correct. Angry overtones may increase the persuasiveness of arguments, for instance because anger is associated with certainty (Lerner and Keltner, 2001), which often increases persuasion (Karmarkar and Tormala, 2010). There is indeed some evidence that a source’s angry expressions can influence the attitudes of a target (Van Kleef et al., 2014). However, it should also be noted that this direct conformity-increasing effect was not replicated in Study 2, where the effect of anger expressions on conformity depended on both the initial level of deviance and the potential instrumentality of conformity in securing acceptance. Future studies may examine these issues into more detail.

Although we used linear modeling to test our hypothesis, it is interesting to consider to what extent the power function predicted by SIT (Latané, 1981) may provide a better description of our data. Additional analyses (not reported) revealed that SIT’s power curve only significantly improved the model fit for the angry reactions manipulation checks in both studies. Thus, consistent with the results from the previously described meta-analysis by Bond (2005), the added complexity of SIT’s power curve was not needed to describe the data. This may be due to the relatively small effect sizes observed here, which yielded insufficient resolution to fit the SIT curve. More realistic settings, where the effects of emotional expressions are undoubtedly stronger than in the simulated interactions studied here, may thus yield different conclusions. Alternatively, the range of angry reactions (0–4) may have been too narrow to show the gradually smaller effects of subsequent angry reactions. Awaiting further research into this direction, we provisionally conclude that the positive relation between angry reactions and felt rejection is best described as linear.

Finally, although using a simulated interaction paradigm affords the high experimental control that is needed to carefully study the relation between the number of angry reactions, rejection, and conformity, the substantial number of participants who doubted the veracity of the simulated interactions also shows that such a paradigm is prone to arouse suspicion in participants. This is an important limitation because it implies that some participants who did not spontaneously express such doubts in the open-ended questions, and were therefore left in the sample, may actually still have had some suspicion. These participants are unlikely to have perceived the situation as social, which may have led them to simply discount the reactions from the other ‘participants,’ thereby reducing the impact of our manipulations. Having some suspicious participants in the sample would therefore render our tests conservative, which means that it is likely that the effects of angry reactions on rejection and conformity that we found here are stronger in a more realistic setting.

In sum, we have shown that deviant individuals feel increasingly rejected as more people react with anger to their deviance, and we have shown that this felt rejection generally undermines conformity. Motivated by a desire to restore the individual freedom of choice, or anti-social tendencies triggered by feeling rejection, this anti-conformity may undermine group functioning. Yet, our analysis also illustrates that this anti-conformity following angry reactions and felt rejection may be overcome depending on two critical contextual factors: the initial level of deviance and the potential instrumentality of conformity for gaining acceptance. In showing these relations, we have demonstrated the harmful effects of reacting with anger to deviance, but also shed some light on the conditions under which angry reactions may be effective in resolving the threat to group functioning posed by deviance. Thus, echoing Van Kleef et al. (2011) observation of emotional influence more generally, these findings show that angry reactions to deviance are a tool to handle with care.

## Conflict of Interest Statement

The authors declare that the research was conducted in the absence of any commercial or financial relationships that could be construed as a potential conflict of interest.

## Footnotes

1 Participants were assumed to doubt the veracity of the procedure if they used one or more of the following words to comment on the situation in their response to the final open-ended question (see ‘Manipulation Checks’): ‘fake’ [*nep*], ‘not real’ [*geen echte*], ‘doubt’ [*twijfel*], ‘don’t believe’ or similar [*ongeloofwaardig*, *geloof niet*, *geloof niks*], or ‘programmed’ [*geprogrammeerd*] (*N* = 34). All remaining open-ended responses were read by the first author. Participants were excluded if (a) they directly (e.g., “I think I haven’t really discussed with other people.”) or indirectly (e.g., “Real people don’t type correct sentences”) stated that they thought their peer’s statements were not coming from real participants (*N* = 17), or (b) correctly guessed the purpose or hypothesis of the study (*N* = 2). Two more participants were excluded because they wrote “The fact that in this room, no four people are participating in the same discussion, makes this quite a strange story” and “I think it’s quite a coincidence that all opinion differed from mine, and that I get questions about how excluded I feel.” One participant was excluded for writing “blabla” when asked to comment on his/her opinion in the group discussion, suggesting that this participant did not take the situation seriously.

2 In all analyses, majority size and angry reactions were entered as unstandardized predictors. As a result, the reported βs and ORs indicate the change (in SD, and in odds, respectively) in the dependent variable that is expected when one member is added to the majority, or when there is one more angry reaction. All other predictors were standardized prior to modeling, and the associated βs and ORs have their regular interpretation.

3 We also fitted the power functions predicted by SIT, but found that this only improved the prediction of the angry reactions manipulation checks. Following Bond (2005) and our own prediction (H1), we therefore focus on the simpler, linear models in the remainder of the paper. We return to this issue in the Section “General Discussion.”

4 The same procedure was used as in Study 1. Participants were excluded if they used any of the words listed in Footnote 1 to describe the study (*N* = 3). The first author then read all remaining open-ended responses. Excerpts from the statements that were coded as indicating doubt about the veracity of the procedure (*N* = 7) are: “appears to be a fake-study”; “don’t really think I was talking to real people”; “‘something went wrong’ in the decision round”; “the discussion didn’t continue, which was probably intentional”; “I wouldn’t be surprised if this was a set-up”; “it didn’t feel as if these were really fellow students”; and “the ‘other participants’ answers’ were childish and not very convincing.” One more participant was excluded because this participant commented that the slider, which was used as the initial opinion measure, was not working.

5 The study originally had a Majority Size (2 vs. 3) × Angry Reactions (0–3) × Decision Context (Private vs. Public) between-subjects design. Due to a programming error in the conditions with a majority size of two (2|A), the simulated group members disagreed with each other in these conditions when the norm was ‘more books’ (i.e., one group member argued for more books; the other for more journals). Thus, these conditions did not represent the intended majority influence situation, and were therefore dropped from the design.

6 An anonymous reviewer alerted us that research with similar manipulations has shown that it may also trigger a motive for accuracy. Although our dependent measure (a vote on the preferred percentage of research articles in the study materials) did not include an objectively accurate answer, and this motive is therefore unlikely to have produced our results, it is important to be aware of this explanation in future research.
